# Antibody Subclass and Glycosylation Shift Following Effective TB Treatment

**DOI:** 10.3389/fimmu.2021.679973

**Published:** 2021-07-05

**Authors:** Patricia S. Grace, Sepideh Dolatshahi, Lenette L. Lu, Adam Cain, Fabrizio Palmieri, Linda Petrone, Sarah M. Fortune, Tom H. M. Ottenhoff, Douglas A. Lauffenburger, Delia Goletti, Simone A. Joosten, Galit Alter

**Affiliations:** ^1^ Ragon Institute of Massachusetts General Hospital, Massachusetts Institute of Technology, and Harvard University, Cambridge, MA, United States; ^2^ Department of Immunology and Infectious Disease, Harvard School of Public Health, Boston, MA, United States; ^3^ Department of Biomedical Engineering, University of Virginia, Charlottesville, VA, United States; ^4^ Department of Internal Medicine, University of Texas Southwestern Medical Center, Dallas, TX, United States; ^5^ Clinical Department, National Institute for Infectious Diseases (INMI), IRCCS L. Spallanzani, Rome, Italy; ^6^ Department of Epidemiology and Preclinical Research, National Institute for Infectious Diseases IRCCS (INMI) L. Spallanzani, Rome, Italy; ^7^ Department of Infectious Disease, Leiden University Medical Center, Leiden, Netherlands; ^8^ Department of Biological Engineering, Massachusetts Institute of Technology, Cambridge, MA, United States

**Keywords:** antibodies, tuberculosis, IgG4, Fc-glycosylation, TB therapy

## Abstract

With an estimated 25% of the global population infected with *Mycobacterium tuberculosis* (*Mtb*), tuberculosis (TB) remains a leading cause of death by infectious diseases. Humoral immunity following TB treatment is largely uncharacterized, and antibody profiling could provide insights into disease resolution. Here we focused on the distinctive TB-specific serum antibody features in active TB disease (ATB) and compared them with latent TB infection (LTBI) or treated ATB (txATB). As expected, di-galactosylated glycan structures (lacking sialic acid) found on IgG-Fc differentiated LTBI from ATB, but also discriminated txATB from ATB. Moreover, TB-specific IgG4 emerged as a novel antibody feature that correlated with active disease, elevated in ATB, but significantly diminished after therapy. These findings highlight 2 novel TB-specific antibody changes that track with the resolution of TB and may provide key insights to guide TB therapy.

## Introduction

Tuberculosis (TB) continues to be one of the leading causes of death by infectious disease globally, and while the development of new protective vaccines continues to be a critically important goal for the fight against TB disease (1, 2), detecting active TB (ATB), the TB state with the greatest likelihood of spreading *Mtb*, for immediate treatment could profoundly prevent TB spread (3–5). Current immune-based diagnostics, including the tuberculin-skin-test (TST) or the interferon-gamma release assay (IGRA), can detect individuals with TB but cannot distinguish individuals with ATB from latent TB infection (LTBI), which accounts for ~95% of world cases, therefore limiting the ability to identify disease that requires immediate treatment (6, 7). Furthermore, current immune diagnostics cannot distinguish those who have successfully completed therapy from those with ATB and actively replicating *Mtb*.

Given the heterogenous manifestation of disease in individuals exposed to *Mtb*, it is not surprising that immune responses to *Mtb* are also heterogenous. And in humans, features of the immune response such as numbers of circulating NK cells (8), neutrophils (9), B cells (9, 10) and T cells (9) have been observed to differ in TB-diseased individuals depending on their disease severity and clearance of replicating *Mtb* following treatment. Phenotypic differences in the T cell response to TB have also been shown to associate with disease severity, with higher frequencies of proliferating and TH1-cytokine producing CD4 T cells observed in ATB compared to LTBI (11–14); these T cells diminish from circulation following therapy (15, 16). In addition, inflammatory signatures that include type I interferon, captured through whole-blood RNA sequencing, demonstrate a strong association with ATB disease, which also diminishes with treatment (9, 17).

Antibody-based measures are attractive alternatives to cellular measures of disease activity; these disease-specific immune responses are easily and directly captured from serum in an antigen specific manner. Measuring antibodies in sera is also technically simple and relatively rapid when compared to the cumbersome and variable measures of cellular immune responses. Importantly, a single antibody molecule measures both antigen-specificity within the variable domain (Fab) and inflammatory state of the disease within the constant domain (Fc) (18). Alterations in disease-specific IgG properties including antigen specific titers (19, 20), isotype switching (21), and glycosylation (22–26) provide insights into disease relapse or severity across diseases ranging from cancer (27, 28), autoimmunity (29, 30), and infections (20, 31). While changes in disease-specific titers do not always reflect changes in disease activity (32–35), alterations in disease-specific Fc-properties provide critical qualitative insights into disease activity. Given that changes in IgG Fc-profiles also track with altered Fc-effector function, unique humoral markers of disease activity may also provide additional insights into the mechanism(s) of enhanced disease control and even elimination. Along these lines, recent studies of TB-specific antibodies highlight the disease discriminatory activity of IgG Fc-glycosylation features (36, 37) that may point to unexpected mechanisms of anti-microbial control (36, 38–42).

Recent data indicate that B cells change not only in number but also phenotype and function during TB disease and after treatment (10). While decreased TB-specific IgG titers have been noted in several studies following TB treatment (43, 44), it remains unclear whether antibody Fc-profiles also shift with treatment. Humoral profile shifts could provide insights into long-term immunity after successful *Mtb* clearance and point to markers of TB treatment success. Thus, in this study we aimed to determine how the humoral immune response to *Mtb* differed among the TB states: LTBI, ATB, and txATB. We profiled the TB-specific humoral immune responses in the serum of individuals previously profiled for B cell phenotype and function (10) using a systems serology approach (45). These measures included total serum antibody titers, antigen-specific antibody titers, and antibody-mediated functional responses in human cells. And in light of our recent study of IgG glycans in LTBI/ATB discriminatory model performance, which found IgG-Fc glycans discriminated better compared to whole IgG and Fab-glycans (37); we focused on IgG-Fc glycans in the humoral profiling of this cohort. We observed significant differences in IgG-Fc glycosylation across individuals with LTBI and ATB disease, consistent with earlier observations in IgG from independent cohorts of LTBI and ATB individuals (37). Additionally, we found enrichment of TB-specific IgG4 among ATB individuals. Strikingly, these same antibody features distinguished ATB from txATB, suggesting that IgG4, in addition to IgG-Fc glycosylation, may also be a marker of ongoing inflammation and the ATB disease activity.

## Methods

### Study Subjects

Sample collection was approved by the ethical committee of INMI, approval number 72/2015; informed written consent was obtained before collection. ATB was confirmed *via Mtb* sputum culture and patients were enrolled within 7 days of starting the TB treatment (isoniazid, rifampicin, ethambutol and pyrazinamide for 2 months, followed by isoniazid, rifampicin for 4 additional months (46). TxATB subjects were patients who completed a 6-month course treatment for culture-positive pulmonary TB and were culture-negative at 2 and 6 months of therapy. LTBI subjects were mainly contacts recently exposed (within the previous 6 months) to smear-positive ATB patients with positive QuantiFERON TB Gold In tube (QFT-IT) (Quiagen, Germany) but without symptoms or radiological signs of ATB. Healthy donors were QFT-IT- and HIV^-^ individuals not undergoing immunosuppressive drug treatments. Serum samples were collected in heparin tubes. An additional 10 healthy HIV^-^ donors from the Greater Boston, Massachusetts area were recruited by Ragon Institute of MGH, MIT, and Harvard for serum assay controls. Blood samples were collected in ACD tubes from different donors in the Boston-area, for the isolation of Neutrophils for ADNP assays. NK cells used for assessing antibody function were derived from buffy coats of healthy HIV- donors collected by the MGH Blood Donation Bank. All study participants for additional healthy negatives and primary cell isolation gave written, informed consent, approved by the institutional review boards at Massachusetts General Brigham Hospital.

### Total Immunoglobulin Quantification

Total quantities of IgG1, IgG2, IgG3, IgG4, IgM, and IgA were determined in serum samples diluted 1:15,000 and used the MILLIPLEX^®^ MAP Human Isotyping Magnetic Bead Panel (Sigma-Millipore HGAMMAG-301K) to quantify total immunoglobulins.

### Antigens for Antibody Profiling

*Mtb* antigen used to profile antibody responses included: PPD (Staten Serum Institute), recombinant (rec.) Ag85A and Ag85B combined in a 1:1 ratio (BEI Resources: NR-14871 and NR-4870), rec. ESAT6 and CFP10 combined in a 1:1 ratio (BEI Resources: NR-14868 and NR-49425), rec. GroES (BEI Resources: NR-14861), rec. glcB (provided by T. Ottenhoff), rec. HspX (BEI Resources; NR-49428). Non-TB infectious antigens included Influenza-HA antigen represented by a mixture of 7 recombinant HA antigens: H1N1-A/Brisbane/59/2007, B/Florida/4/2006, B/Malaysia/2506/2004, H1N1-A/Solomon Island/3/2006, H3N2-A/Wisconsin/67/X-161/2005, H3N2-A/Brisbane/10/2007 and H1N1-A/New Caledonia/20/99 (Immune Technology); tetanus toxin (Mass Biologics Lp1099p); and rec. pp65 for CMV (Abcam, 43041).

### Custom Luminex Assay for Ag-specific Titer Determination

Multiple unique Luminex MagPlex carboxylated bead regions (Luminex) were coupled with the above-mentioned antigens to determine the antigen-specific titers present in the cohort serum samples. Serum samples were diluted 1:30, 1:100, 1:300, and 1:1000, and 1:3000 to generate an area under the curve (AUC) measurement using the detection reagents total IgG, IgG1, IgG2, IgG3, IgG4, IgM, IgA1, and IgA2 (Southern Biotech).

### Antibody Dependent Cellular Phagocytosis (ADCP)

Biotinylated PPD was used to coat 1mm Neutravidin labeled yellow-green, fluorescent beads. Immune complexes were formed by combining PPD-beads, and combined with serum samples diluted 1:30, 1:100, 1:300, 1:1000 in PBS and incubated at 37C for 2hrs. Complexes were washed, 2x10^4^ THP1 cells were added per well of 96-well plates and incubated for 1hr at 37C. Samples were then washed and fixed for analysis of bead uptake on an iQue Screener. Phagocytic scores were calculated as previously described (36) across sample dilution series and were used to calculate AUC.

### Antibody Dependent Neutrophil Phagocytosis (ADNP)

Neutrophils were isolated from healthy donor blood collected in ACD tubes as previously described (36). Complexes were formed and incubated with isolated neutrophils as described for the ADCP above. After incubation, samples were stained with CD66b and fixed with 4%PFA. Bead phagocytosis was measured as described above, and the enrichment of neutrophils was confirmed with CD66b staining (BioLegend 305112). Phagocytic scores were determined as in ADCP across a serum dilution series ranging from 1:30 to 1:1000 and used to calculate Phagocytic Score AUC.

### Antibody Dependent NK Cells Activation

ELISA plates were coated with 50mL of 2μg/mL PPD overnight at 4C. Coated plates were blocked with 5% BSA for 1hr at RT and washed 3x with PBS before 50μL of diluted serum (1:30, 1:100, 1:300, 1:1000) was added to each well. Serum dilutions were incubated 2hrs at 37C on antigen-coated plated and washed prior to adding 5x10^4^ NK cells per well, isolated from healthy HIV^-^ donor buffy coats by RosetteSep (Stem Cell 15065). CD107a-BV605 (BioLegend 328634), 5μg/ml brefeldin A (BioLegend 420601), and 0.7μl/mL GolgiStop (BD 554724) were also added to each well and incubated for 5hrs at 37C. Following this incubation, NK cells were surface-stained with CD16-BV785 (BioLegend 302046), CD56-PE-Cy7 (BD 335791), and CD3-APC-Cy7(BioLegend 300426). An intracellular stain was then performed using Perm/Fix Solution (BD 554714) with IFNγ-PE (BioLegend 506507) and anti-MIP1β-BV421(BD 562900). Samples were fixed with 4% and NK cell activation was analyzed on the iQue Screener. AUC frequencies of NK cells bearing CD107a, expressing IFNγ and MIP1β across, were derived from the signal across the dilution series tested in the donor cells.

### Glycan Analysis of IgG-Fc

IgG was isolated from serum samples by incubating 10μL of serum diluted 1:20 in PBS with 25μL protein G beads (Millipore, Catalog #LSKMAGG10); the serum and beads were mixed at 4C for 16hrs. Excess serum protein was washed, and IgG-bound beads resuspended in digestion buffer containing 1uL IdeZ (NEB Catalog #P0770S) and IgG was digested at 37C for 2hr to remove Fab. IgG-Fc still bound to magnetic protein G beads were pelleted and washed on a magnet to separate Fc from Fab. Glycans from IgG-Fc were cleaved, enriched, and labeled with APTS according to manufacturer specifications in the Glycan Assure Kit (ThermoFisher A28676). To immune-precipitate and analyze antigen-specific IgG-Fc glycosylation, streptavidin-coated magnetic beads (NEB Cat# S1420S) were coated with biotinylated-PPD, as described above for ADCP. Antigen-coated beads were incubated with 300μL of serum at 4C for 16hrs. Excess protein was washed off the beads with PBS and Fc of the antigen-bound IgG was cleaved with IdeZ as described above. Supernatants were taken from this IdeZ reaction for glycan cleavage and staining according to the Glycan Assure protocol. Samples were run with a LIZ 600 DNA ladder in Hi-Di formamide (Thermo Fisher 4408399) on an ABI 3130XLl DNA sequencer. Data were analyzed using ThermoFisher Glycan Assure Analysis software; peaks were assigned based on migration of standards of known glycans and peak area was calculated. The measured peak areas per sample were totaled to report a relative frequency of each glycan structure identified.

### Data Visualization and Analysis

Univariate data visualization and statistical analysis were performed using GraphPad Prism (Version 8.3.1). For multivariate analysis, MATLAB computing environment (version 2018b, Mathworks, Natick, MA) was used, supported by the Statistics and Optimization toolboxes, as well as the third party PLS toolbox (Eigenvector Research, Inc, Manson, WA). Spearman network visualizations were performed using Cytoscape (version 3.6.0).

### Identification of TB Signatures With LASSO and OPLSDA

Computational analysis was used to build classification models that identify key features that most effectively resolved pairs of the LTBI, ATB and txATB states. These classification models were built using previously described methods (25, 45) combining (i) Least Absolute Shrinkage and Selection Operator (LASSO) method (47), for feature selection, and (ii) classification using the LASSO-selected features. For LASSO selection, a previously described nested cross validation framework was used to validate the robustness of the classification model (48). Orthogonalized Partial Least Square Discriminant Analysis (OPLSDA) (49, 50) was used to visualize LASSO-selected variables and assess their predictive ability for classifying TB states (**Figures 2**, **3**, **4A, B**). These input variables were centered and scaled to a standard deviation of 1. PLSDA models consisting of two LVs were constructed and then orthogonalized to condense the Y-block variance (group separations) into the first Latent Variable (LV1). LV1 captures the variance in features that are in the direction of the pairwise separation of the groups, while LV2 describes the variation orthogonal to this predictive component. To assess each model, 5-fold cross validation (CV) was performed on the data (100 random 5-fold cross validation). To assess model significance, permutation test was performed on the cross validated models by randomly shuffling the labels. The OPLSDA models performed significantly better than random with CV Wilcoxon p values of lower than 2E-3 across pairwise group comparisons. Variable Importance in Projection (VIP) scores were calculated (51) to rank the importance of each variable in the projection of the PLS model. To emphasize the direction of the contribution of each variable, negative and positive signs were added to VIP scores to indicate negative and positive Loadings of each variable on LV1.

### Construction of the Correlation Network of the LASSO-Selected Features

Spearman correlation of the LASSO-selected Fc features to all original 78 TB-specific antibody features were calculated. Each node is a feature and the thickness of the edges between nodes is proportional to their correlation coefficients. The p-value depicting the significance of these correlations were corrected for multiple comparisons (Benjamini-Hochberg q-value < 0.05, testing the hypothesis of zero correlation). Only correlations with corrected p-values<0.05 were included.

### Three-Way PLSDA Model

The LASSO-selected features from the three pair-wise group comparisons were pooled (total of 8 features) and a PLSDA model was developed to separate the three groups of LTBI, ATB and txATB. This model was not orthogonalized to better capture and visualize the pairwise group differences. The two-dimensional loadings on LV1 and LV2 were overlaid on the scores plot.

## Results

### LTBI and ATB Plasma Have Distinct Profiles of Fc-Glycosylation and TB-Specific IgG Subclass

We previously described the biophysical and functional features of purified IgG found in LTBI and ATB from cohorts of individuals from South Africa and US/Mexico (36, 37). However, whether these differences persist, or differ following successful antibiotic treatment remains unclear. Thus, here we aimed to define the impact of therapy on shaping the TB-specific humoral immune in a cohort of LTBI (n=21), ATB (n=20), and txATB (n=23) from Italy, with an additional group of healthy control individuals (n=17) from Italy and the USA (**Table 1**). Similar levels of circulating, non-antigen-specific IgM, total IgG and the subclasses IgG1, IgG2, Ig3, and IgG4 were observed in the serum of healthy, LTBI, ATB, and txATB individuals (**Supplemental Figure 1A**). In contrast, total IgA titers were significantly elevated in ATB compared to LTBI and txATB (**Supplemental Figure 1A**), consistent with previous observations in TB (52, 53). Additional differences in antigen-specific isotype (**Supplemental Figures 1B–E**), subclass (**Supplemental Figure 2**), total IgG-Fc glycosylation (**Supplemental Figure 3**), and Fc-mediated functional antibody responses in monocytes, neutrophils, and NK cells (**Supplemental Figure 4**) were noted across groups. The differences observed across this collection of measures suggested distinct humoral profiles existed not only in LTBI and ATB, but also txATB.

**Table 1:** Cohort Demographics.

		LTBI	ATB	txATB	Controls
Individuals:		21	20	23	17
Age, (median +IQR):		31 (21-77)	35 (23-67)	37 (17-70)	31(23-57)
Gender, Females:		15 (71%)	2 (10%)	15 (65%)	10 (59%)
BCG Vaccinated:		9 (41%)	19 (95%)	15 (65%)	5 (29%)
Mos. post treatment (median IQR):				8 (1-72)	
Origin, n (%):	West Europe:	12 (57%)	1 (5%)	10 (43%)	14 (825)
	East Europe:	5 (24%)	15 (75%)	7 (30%)	2 (12%)
	Asia:		1 (5%)	1 (4%)	
	Africa:	2 (10%)	2 (10%)	3 (13%)	
	South America:	2 (10%)	1 (5%)	2 (9%)	1 (6%)

**Figure 1:**
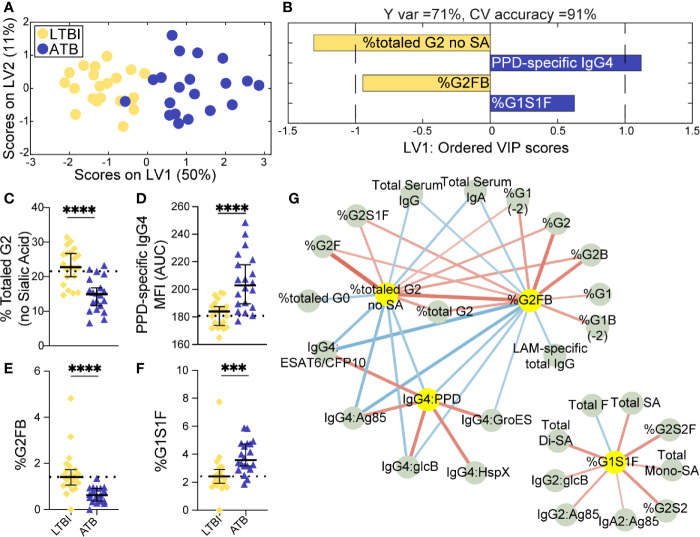
Fc-glycosylation and TB-specific IgG subclass distinguish LTBI and ATB individuals. An orthogonalized PLSDA ***(***OPLSDA) model was created based on four LASSO-identified antibody features that discriminate LTBI from ATB **(A, B)**. Latent variable 1 (LV1) explains 71% of Y variance in the direction of LTBI and ATB separation. 5-fold cross validation (CV) was performed, resulting in 91% CV accuracy. The model significantly outperformed models based on shuffled group labels (permutation testing, Wilcoxon p=2E-5) **(A)** PLSDA scores plot depicts model separation of LTBI (n = 21, yellow dots) and ATB (n = 20, blue dots). LV1 and LV2 account for 50% and 11% of the variability of the input features. **(B)** Variable Importance in Projection (VIP) scores plot of top features providing the greatest resolution of LTBI and ATB in rank-order. Directions of the bars signify loadings on LV1 and colors represent the disease groups in which measures were enriched. Pairwise comparison of LTBI (n = 21, yellow diamonds) and ATB (n = 20, blue triangles) individuals **(C)** The frequencies of totaled G2 structures without sialic acid on IgG-Fc of LTBI and ATB individuals. **(D)** AUC of PPD-specific IgG4 titers. **(E)** percentage of G2FB glycan on IgG **(F)** percentage of G1S1F glycan on IgG. Univariate plots **(C-F)** show median and interquartile range of each LASSO-selected measure; statistically significant differences between LTBI and ATB groups calculated using Mann-Whitney test: ***p < 0.0005 and ****p < 0.0001. The dotted lines represent median of healthy controls individuals tested. **(G)** Correlation analysis depicts other features that are positively (red lines) or negatively (blue lines) correlated with these four key features selected with LASSO (highlighted in yellow). The color intensity and width of the edges between nodes are proportional to the significance of correlation coefficients after correcting for multiple comparisons (Benjamini-Hochberg q-value < 0.05, testing the hypothesis of zero correlation). Only correlations with corrected p-values < 0.05 were included.

Given the univariate differences across this cohort, we sought to identify the humoral features of our dataset, which could best discriminate subsets of TB diseased individuals by applying a conservative multivariate analytic method to define a minimal set of distinguishing humoral features. We first aimed to determine whether LTBI and ATB were fully resolvable using antibody features as had been previously observed (36), and further, whether a minimal set of antibody-features could be identified, which resembled previous antibody profile differences. A least absolute shrinkage and selection operator (LASSO) (47) was first applied to reduce the number of features in our highly correlated dataset (Data Sheet 1), to avoid overfitting, and define a minimal set of these features that could discriminate the LTBI and ATB disease states. Using PLSDA for visualization, clear separation was observed between the LTBI and ATB antibody profiles (**Figure 1A**). Moreover, as few as 4 of the 78-total Fc-features selected by LASSO could resolve LTBI and ATB individuals, providing 91% cross-validation (CV) accuracy (**Figure 1B**); these included totaled di-galactosylated (G2); the di-galactosylated, fucosylated, and bisected (G2FB) structure; PPD-specific IgG4 levels; and singly-galactosylated, sialylated, fucosylated (G1S1F) structures.

Univariate plots of the LASSO-selected features revealed statistically significant differences in all 4 features when comparing LTBI and ATB groups (**Figures 1C–F**). Specifically, the levels of totaled G2 (**Figure 1C**) and the G2FB structure (**Figure 1E**) on total IgG-Fc were enriched in LTBI. While PPD-specific IgG4 levels and the G1S1F structure were enriched in ATB IgG-Fc (**Figures 1D, F**). The differential IgG-Fc galactosylation seen across LTBI and ATB of this cohort is consistent with our previous observations in cohorts of LTBI and ATB samples from South Africa and the US/Mexico (36). However, the subclass enrichment of IgG4 antibodies in ATB represents a novel observation in TB disease. The coincidence of IgG4 and G1S1F on total IgG-Fc glycans of ATB is reminiscent of previous studies of subclass specific Fc-glycosylation, which found an enrichment in IgG4 of healthy individuals (54) and an elevation of G1S1F on IgG4 in patients with IgG4-related disease (55).

Given that the LASSO/PLSDA model selects features that solely account for the greatest variance across the antibody profiles being compared, additional distinctive antibody features that are correlated with the LASSO-selected features are not highlighted in this analysis. To explore the additional humoral features of our dataset that distinguished LTBI and ATB, we next generated a Spearman correlation network of the LASSO-selected features to highlight the relationship of this minimal set of features with the remaining 74 features measured (**Figure 1G**). Two networks emerged from this analysis: a large network linking the two LTBI enriched features (G2 and G2FB) linked *via* negative correlations to the ATB enriched IgG4 signature (**Figure 1G**, right), and a smaller second network consisting of features correlated with the ATB-associated G1S1F feature (**Figure 1G**, left). Importantly, IgG4 titers against multiple TB antigens, including intracellular TB proteins HspX and GroES, were co-correlated, pointing to a shift to IgG4 responses in ATB. Additionally, di-galactosylated features enriched in LTBI were linked to several galactosylated structures, reinforcing an overall elevated galactosylation profile in LTBI. Finally, total serum IgG and IgA levels, found to be elevated in ATB (**Supplemental Figure 1A**), were inversely correlated to the glycan features elevated in LTBI profile. These networks link qualitative/quantitative changes in the humoral profiles between LTBI and ATB states with the LASSO-selected features and highlight the unique enrichment of *Mtb*-specific IgG4 responses. Furthermore, depletion of IgG4 resulted in increased antibody effector function in neutrophils and NK cells (**Supplemental Figure 5**), pointing to IgG4 as a mechanistic player in dampening antibody function, as previously shown in HIV (56) and cancer (57). In summary, we find a critical recapitulation of glycan features of latency in an Italian cohort, highlighting the universal presence of this biomarker of ATB, that includes IgG4 levels, perhaps previously overlooked due to the purification methods used in our original study. These data reinforce a set of qualitative antibody Fc-features that discriminate LTBI from ATB.

### Treatment of ATB Correlates With Reduced TB-Specific IgG4 Titers and Inflammatory Glycan Signatures

Treatment of ATB and the resolution of replicating *Mtb* has been linked to the resolution of inflammatory cytokines (9), shift in T cell phenotypes (15) and NK cell abundance (8) in the blood. Along these lines, previous cellular profiling of this cohort of individuals indicated that B cells were less proliferative and produced fewer antibodies in ATB, while B cells functioned normally following treatment (10). Given these previously observed differences following TB treatment, we next aimed to determine whether a minimal set of humoral features could distinguish txATB from ATB and mirror the recovered B cell responses found in txATB individuals.

**Figure 2:**
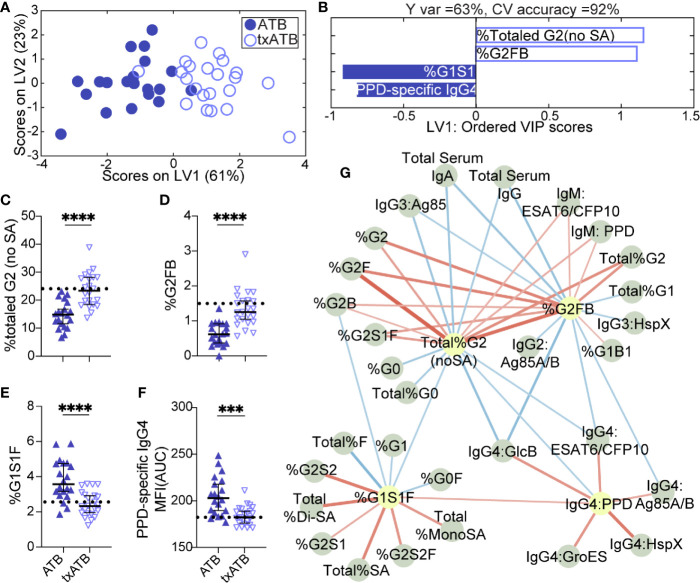
Fc-glycosylation and TB-specific IgG subclass distinguish ATB and txATB individuals. An OPLSDA model was constructed using LASSO-identified antibody features as input and txATB and ATB group separation as output **(A, B).** LV1 explains 63% of the Y variance in the direction of the txATB and ATB. 5-fold cross validation resulted in 92% CV accuracy. The model performed significantly better than models based on shuffled group labels in permutation testing (Wilcoxon p=1E-5). **(A)** OPLSDA scores plot depicts model separation of ATB (n = 20, blue dots) and txATB (n = 23, periwinkle open dots). LV1 and LV2 account for 61% and 23% of the variability in the input features. **(B)** VIP scores plot of top features providing the greatest resolution of ATB and txATB in rank-order. Directions of the bars signify loadings on LV1 and colors represent the disease groups in which measures were enriched. Pairwise comparison of ATB (n = 20, blue triangles) and txATB (n = 23, periwinkle open triangles) individuals **(C)** The frequencies of totaled G2 structures without sialic acid on IgG-Fc of ATB and txATB individuals. **(D)** percentage of G2FB glycan on IgG **(E)** percentage of G1S1F glycan on IgG. **(F)** AUC of PPD-specific IgG4 titers. Univariate plots **(C–F)** show median and interquartile range of LASSO-selected features and statistically significant differences between ATB and txATB groups calculated using Mann-Whitney test: ***p < 0.0005, and ****p < 0.0001. The dotted lines represent median of healthy controls **(G)** Correlation analysis depicts other features that are positively (red lines) or negatively (blue lines) correlated with these four key features selected with LASSO (highlighted in yellow). The color intensity and width of the edges between nodes are proportional to the significance of correlation coefficients after correcting for multiple comparisons (Benjamini-Hochberg q-value < 0.05, testing the hypothesis of zero correlation). Only correlations with corrected p-values < 0.05 were included.

LASSO was applied to select minimal features that distinguished ATB and txATB, and PLSDA visualization of the selected features provided nearly perfect distinction between the disease states, with 92% cross-validation accuracy (**Figure 2A**). The LASSO-identified features included: total IgG-G2, -G2FB, -G1S1F glycans, and PPD-specific IgG4 (**Figure 2B**). These antibody features point to the resolution of inflammatory Fc-glycosylation with treatment, with elevated G2 (**Figure 2C**) and G2FB (**Figure 2D**) structures on txATB IgG-Fc compared to ATB. In contrast, the frequency of IgG-G1S1F structures was significantly higher in ATB compared to txATB (**Figure 2E**). Finally, PPD-specific IgG4 titers were lower in txATB compared to ATB (**Figure 2F**), with txATB levels of PPD-specific IgG4 equivalent to those found in healthy controls (**Supplemental Figure 2D**). Collectively, these data suggest that inflammatory glycans and PPD-specific IgG4 titers, markers of ATB, are diminished in txATB.

Again, to further probe the dataset for additional shifts in the humoral response related to the LASSO-selected features, we generated a Spearman correlation network between the LASSO-selected features and the remaining features. A single correlated network emerged (**Figure 2G**), including a dense cluster of glycans and TB-specific IgG4 features that all diminished in the setting of treatment. In addition to the network of IgG4 titers that inversely correlated with glycan structures enriched in txATB, IgG2 and IgG3 titers specific for Ag85A/B and HspX features arose in the network and inversely correlated to G2 features. These relationships suggest that in addition to IgG4 titers, individuals with ATB utilize additional IgG subclasses, during persistent infection and exposure to *Mtb* antigens, that likely resolve following treatment. Finally, IgM-specific responses to PPD and ESAT6/CFP10 emerged in this analysis and were linked to G2 levels on IgG-Fc in the txATB group. As the first class of antibody produced in primary antigen exposure (58), the elevation of TB-specific IgM titers in txATB point to a development of novel naïve humoral responses following resolution of replicating *Mtb* (**Supplemental Figure 1B**). Together, these data highlight a significant shift in antibody isotypes following treatment, with an overall reduction of inflammatory IgG-Fc glycans and concomitant contraction of the ATB-specific IgG4 immunity across TB-specific antigens.

### Higher TB-Specific Titers Distinguish txATB From LTBI

**Figure 3:**
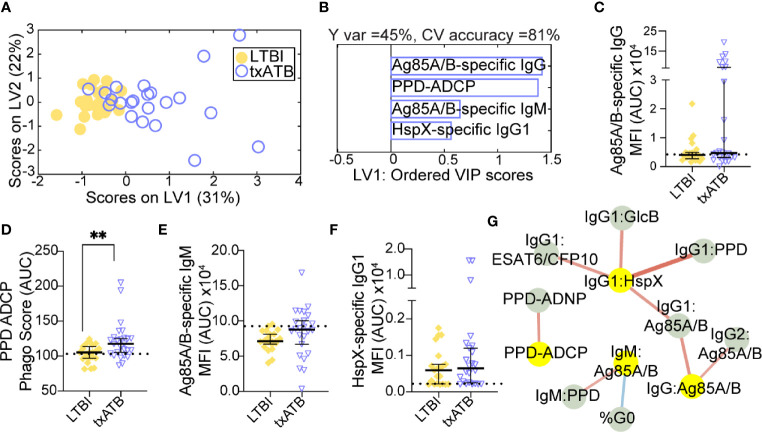
TB-specific IgG titers distinguish LTBI and txATB individuals. An OPLSDA models was constructed using the LASSO-selected features to discriminate LTBI and txATB **(A, B)**. The variance in the direction of separation of LTBI and txATB was condensed on LV1 (Y variance = 45%). 5-fold cross validation was performed, resulting in 81% CV accuracy. Permutation testing was performed, which showed that this model performed significantly better than models based on shuffled group labels (Wilcoxon p=0.002). **(A)** OPLSDA scores plot depicts separation of LTBI (n = 21, yellow dots) and txATB (n-23, periwinkle open dots). LV1 and LV2 account for 31% and 22% of the variability in the input features. **(B)** VIP scores plot of top features providing the greatest resolution of LTBI and txATB in rank-order. Directions of the bars signify loadings on LV1 and colors represent the disease groups in which measures were enriched Pairwise comparison of LTBI (n = 21, yellow dots and diamonds) and txATB (n=23, periwinkle dots and triangles) individuals. **(C)** AUC Ag85A/B-specific total IgG titers. **(D)** PPD-specific ADCP Phago Score **(E)** AUC Ag85A/B-specific IgM titers **(F)** AUC HspX-specific IgG1 titers. Univariate plots **(C–F)** shows median and interquartile range of LASSO-selected features and statistically significant differences of the LASSO-selected features between LTBI and txATB groups calculated using Mann-Whitney test: **p < 0.01. The dotted lines represent median of healthy controls **(G)** Correlation analysis depicts other features that are positively (red lines) or negatively (blue lines) correlated with these four key features selected with LASSO (highlighted in yellow). Edges between nodes are weighted using significant correlation coefficients after correcting for multiple comparisons (Benjamini-Hochberg q-value < 0.05, testing the hypothesis of zero correlation). Only correlations with corrected p-values < 0.05 were included.

The overlapping features that distinguished both LTBI/ATB and ATB/txATB raised the question of whether humoral immunity in txATB and LTBI were largely similar or if humoral immunity could also distinguish these two states. Using LASSO/PLSDA on the humoral profiles, LTBI and txATB could be resolved with 81% cross-validation accuracy (**Figure 3A**). The LASSO selected features were largely enriched in the txATB compared to the LTBI individuals (**Figure 3B**); these included Ag85A/B-specific IgG and IgM titers as well as HspX-specific IgG1 titers (**Figures 3C, E, F**). And while the LASSO-selected TB titers did not reach univariate significance, the amount of antibody-mediated phagocytosis (ADCP) was significantly increased in txATB compared to LTBI (**Figure 3D**). These LASSO-selected features indicate higher antibody levels and function amongst txATB individuals. Moreover, network analysis underscored the prevalence of higher IgG1 titers against multiple TB-specificities in txATB individuals and pointed to the persistence of TB-specific antibodies in this recently treated population (**Figure 3G**). The elevated ADCP activity in txATB was linked to elevated neutrophil phagocytosis (ADNP), highlighting persistent antibody-mediated phagocytic activity following antibiotic treatment. Thus, txATB was distinguishable from LTBI in our multivariate analysis by higher antibody titers and enhanced opsonophagocytic function at the conclusion of treatment.

### Antibody Titer and Glycosylation Distinguish txATB From ATB and LTBI

**Figure 4:**
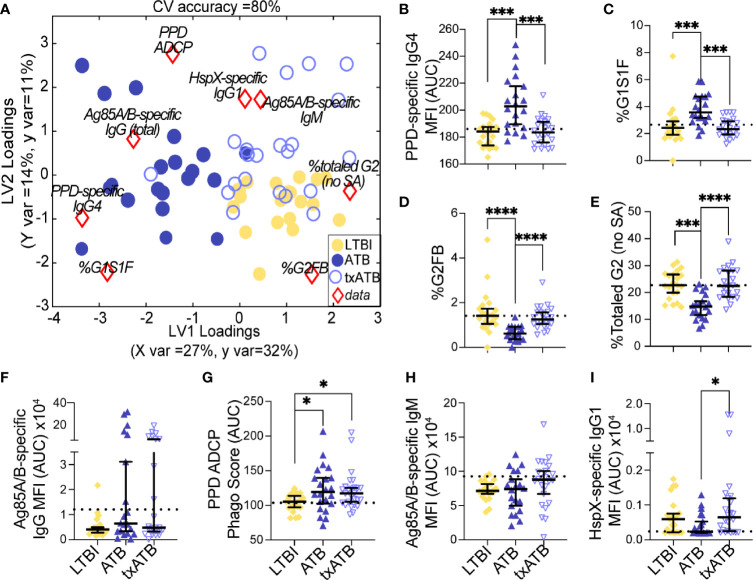
Fc-glycans, TB-specific titers, and isotypes distinguish individuals on the TB spectrum. Three-way comparison of LTBI (n = 21, yellow dots and diamonds), ATB (n = 20, blue dots and triangles), and txATB (n = 23, periwinkle dots and triangles) individuals. **(A)** Using the features described in **Figures 2** **–** **4**, a three-way PLSDA analysis summarizes the comparison of LTBI, ATB, and txATB. The depicted biplot overlays the scores plots of individuals color-coded based on their TB status on the two-dimensional loading plots of the 8 input features (red diamonds). LV1 accounts for 27% of variance in X and 32% of variance in Y, whereas LV2 explains 14% X variance and 11% Y variance. To assess the model performance, 5-fold cross validation was performed resulting in 80% CV accuracy. Permutation testing results showed that this model outperformed 90% of models based on shuffled group labels (Wilcoxon p = 0.1). **(B)** AUC of PPD-specific IgG4 titers. **(C)** frequency of G1S1F glycan on IgG **(D)** frequency of G2FB glycan on IgG **(E)** The frequencies of totaled G2 structures without sialic acid on IgG-Fc. **(F)** AUC Ag85A/B-specific total IgG titers. **(G)** PPD-specific ADCP Phago Score. **(H)** AUC Ag85A/B-specific IgM titers **(I)** AUC HspX-specific IgG1 titers. Univariate plots **(B-I)** show median and interquartile range of the measured values and statistically significant differences between LTBI, ATB, and txATB groups were calculated using Kruskal-Wallis test with Dunn’s multiple comparison test correction: *p < 0.05, ***p < 0.0005, and ****p < 0.0001.

Given the ability of our pairwise models to distinguish TB states, we next aimed to resolve all three TB states simultaneously using the features previously selected by LASSO. Strikingly, this model discriminated between all three states with 80% classification accuracy (**Figure 4A**). ATB was most distinct from LTBI using these features and showed some overlap with txATB. However, interdigitation was observed across LTBI and txATB individuals, pointing to an overlap of humoral profiles in these disease states. LASSO-selected features were superimposed on the PLSDA plot, in the quadrant in which it was enriched (**Figure 4A**). PPD-specific IgG4 and IgG-Fc glycan, G1S1F, were uniquely enriched amongst the cluster of ATB individuals within the PLSDA. A markedly higher level of Ag85-specific IgG was also associated with the ATB cluster. Conversely, enhanced levels of PPD-specific phagocytosis, elevated HspX-IgG, and Ag85-IgM were observed among txATB. Finally, enhanced di-galactosylation was observed on IgG-Fc from LTBI highlighting the importance of titers, function, and glycosylation, many of which were significantly elevated in LTBI and txATB compared to ATB at a univariate level (**Figures 4B–I**).

## Discussion

With our growing appreciation of antibodies in TB immunity (36–39, 59, 60), changes in antibody isotype, subclass, and glycosylation are all emerging as biomarkers specific of disease activity. While many studies have described distinctive antibody features across LTBI and ATB disease, less is known about the changes in humoral immunity following treatment. Using systems serology, we observed significant differences in TB-specific antibody profiles across LTBI, ATB, and txATB, which highlight antibody changes that correlate with the burden of infection. As previously observed (36, 37), we found that LTBI and ATB are marked by distinct IgG-Fc glycosylation patterns, with an enrichment of glycans associated with inflammation in ATB. These observations are in line with an accumulation of inflammatory-glycans observed in antibodies of diseases including HIV (25) and autoimmune disorders (61–63). Importantly, we describe for the first time in txATB individuals an enrichment of G2 structures on IgG-Fc after the successful completion of treatment, pointing to IgG Fc-glycosylation as a marker of reduced replicating *Mtb*. A similar shift in IgG-Fc digalactosylation has been reported in a longitudinal study of IgG-Fc glycans in patients infected with hepatitis B; as treatment progresses and detectable viral DNA decreases, IgG-Fc digalactosylation increases (26). Additionally, we measured PPD-specific IgG-Fc glycans in the serum of a subset of TB diseased individuals from this cohort and found that levels of digalactosylated structures were also lower in ATB compared to LTBI and txATB (**Supplemental Figures 6C, I**). Unlike total IgG-Fc glycan measures, PPD-specific IgG-Fc was enriched for agalactosylated structures in ATB (**Supplemental Figure 6G**), a glycan structure found to be enriched on IgG in other inflammatory diseases (25). Thus, inflammatory Fc-glycans mark disease activity and track with the presence of replicating *Mtb* in ATB individuals.

Beyond antibody glycan changes, humoral comparisons within this cohort pointed to TB-specific IgG4 as a novel humoral marker of TB disease activity. While consistently low in titer, previous studies noted elevated LAM-specific (64) and PPD-specific IgG4 titers (65) in HIV- and HIV+ individuals with ATB, respectively. IgG4 emerges through class-switch late in disease due to its distance on the human IgG locus, and with low Fc-receptor and complement affinity is selected under high antigen-burden when antibody titers are high. In diseases with prolonged antigen exposure and inflammation including parasitic infections (66, 67), chronic *Staphylococcus aureus* (*S. aureus*) infection (68), chronic infectious aortitis (69), melanoma (57), and even following repeated high-antigen dose immunization (56, 70, 71) elevated IgG4 levels have been described. And in the wake of resolving antigen burden, a longitudinal analysis of patients chronically infected with *Brugia malayi*, demonstrated a rapid loss of IgG4 titers, and a preservation of IgG1 responses following treatment (67). Similarly, we observed no change in TB-specific IgG1 titers and a loss of TB-specific IgG4 titers in txATB, pointing to a similar trajectory of IgG4 as TB antigen is eliminated with therapy. And analogous to IgG2 and IgG3 titer declines observed following *Brughia malayi* treatment (67), although not statistically significant, we noted trends of decreased IgG2 and IgG3 titers in txATB compared to ATB in our co-correlate network analysis (**Figure 2G** and **Supplemental Figures 2B, C**). These data suggest that elimination of the antigen results in a shift of antibody subclass selection, with a more dramatic loss of IgG4 antibodies, suggesting that across TB disease, IgG4 may mark a more transient population of antibody-secreting cells that require high-antigenic stimulation to persist, making TB-specific IgG4 an attractive disease-specific marker of treatment success.

The balance of subclass, isotype, and glycosylation within an antibody immune complex can have significant functional consequences for Fc-mediated immune responses. IgG4 may arise to compensate for the pro-inflammatory activity of agalactosylated glycans that accumulate in active inflammatory diseases. In chronic parasitic infections, IgG4 levels are elevated in individuals with asymptomatic parasitic worm infection compared to symptomatic patients, and this IgG4 has been linked to immune-suppressed states (72) and blocking of antibody-mediated hypersensitive responses in basophils (66). IgG4 tend to exhibit enhanced antigen-affinity (73, 74); thus, IgG4 may outcompete binding of functional TB-specific antibodies in immune complexes, thereby diminishing antibody effector activity. And a study focusing on IgG4 biology, using monoclonal antibodies, found that IgG4 antibody could block phagocytic functions of antibodies (57). Moreover, a study comparing Yanomami people and Brazilians of European descent with ATB, found an association of TST anergy and elevated TB-specific IgG4 in the Yanomami people with ATB (75), leading the authors to speculate that the Yanomami developed immune responses to *Mtb* infection that is poorly protective against TB disease. Thus, the presence of IgG4 titers may not only be indicative of high antigen-burden in ATB but might dampen the antibody-mediated functions in TB.

Our findings point to a unique antibody profile in txATB that differs from ATB and LTBI. Both inflammation-associated glycosylation and IgG4 titers found in ATB are diminished upon completion of treatment, providing an attractive set of humoral features to explore more broadly in longitudinal studies tracking treatment success. It should be noted that our co-correlate network analysis in the pairwise comparisons of LTBI/ATB (**Figure 1G**), ATB/txATB (**Figure 2G**), and LTBI/txATB (**Figure 3G**) also highlighted both IgA and IgM related features that highly correlated with the features selected by LASSO/PLSDA models with additional discriminatory potential for the TB disease states studied here. We found significantly elevated total IgA, enriched in ATB, inversely correlated with digalactosylated IgG-Fc levels found in LTBI and txATB, suggesting expanded IgA titers also mark ATB state. Consistent with total IgA expansion in ATB, IgA2 titers specific for Ag85A/B were positively correlated were G1S1F on IgG-Fc (**Figure 1G**). While extensive antigens were not used to characterize the IgA response in this cohort, this observation is consistent with several findings of elevated TB-antigen specific IgA titers in untreated TB (52, 53, 76). Interestingly, PPD-IgM and HspX-IgG1 titers were significantly higher in txATB compared to ATB of this cohort (**Supplemental Figures 1** and **2**) and is consistent with a previous observation of expanded TB-specific antibody titers developing following TB treatment, which tracked with the control of replicating *Mtb* (77). Further studies will be important to identify *Mtb* antigen specificities that expand during therapy, which could be used to track treatment responses.

## Data Availability Statement

The raw data supporting the conclusions of this article will be made available by the authors, without undue reservation.

## Ethics Statement

The studies involving human participants were reviewed and approved by INMI Ethical Committee Approval Number 72/2015. The patients/participants provided their written informed consent to participate in this study.

## Author Contributions

GA and PG conceived and designed the experiments. PG, AC, and LL performed the experiments. Data was analyzed by PG and SD. Multivariate analysis and modeling were performed by SD. Clinical cohort was established and collected by DG, FP, and LP. The manuscript was written by PG, SD, and GA with contributions from SJ, TO, DG, SF, and DL. All authors contributed to the article and approved the submitted version.

## Funding

This work was supported by the Ragon Institute (to GA), the SAMANA Kay MGH Research Scholar Program (to GA), NIH T32 AI007061 (to PG), and Italian Ministry of Health Ricerca corrente, Linea 4 (to DG).

## Conflict of Interest

GA is a co-founder of SeromYx Systems Inc. GA’s interests were reviewed and are managed by Massachusetts General Hospital and Partners HealthCare in accordance with their conflict of interest policies.

The remaining authors declare that the research was conducted in the absence of any commercial or financial relationships that could be construed as a potential conflict of interest.

The handling Editor has declared past collaborations with the authors TO and SJ within the last two years.

## References

[1] Ottenhoff THM Kaufmann SHE. Vaccines Against Tuberculosis: Where Are We and Where do We Need to Go? PloS Pathog (2012) 8(5):e1002607–e1002607. 10.1371/journal.ppat.1002607 22589713 PMC3349743

[2] Abu-Raddad LJ Sabatelli L Achterberg JT Sugimoto JD Longini JIM Dye C. Epidemiological Benefits of More-Effective Tuberculosis Vaccines, Drugs, and Diagnostic. Proc Natl Acad Sci - PNAS (2009) 106(33):13980–5. 10.1073/pnas.0901720106 PMC2720405 19666590

[3] Lönnroth K Corbett E Golub J Godfrey-Faussett P Uplekar M Weil D. Systematic Screening for Active Tuberculosis: Rationale, Definitions and Key Considerations [State of the Art Series. Active Case Finding/Screening. Number 1 in the Series]. Int J Tuberculosis Lung Dis (2013) 17(3):289–98. 10.5588/ijtld.12.0797 23407219

[4] Marks GB Nguyen NV Nguyen PTB Nguyen T-A Nguyen HB Tran KH. Community-Wide Screening for Tuberculosis in a High-Prevalence Setting. N Engl J Med (2019) 381(14):1347–57. 10.1056/NEJMoa1902129 31577876

[5] Lonnroth K Migliori GB Abubakar I D'Ambrosio L de Vries G Diel R. Towards Tuberculosis Elimination: An Action Framework for Low-Incidence Countries. Eur Respir J (2015) 45(4):928–52. 10.1183/09031936.00214014 PMC4391660 25792630

[6] Pai M Denkinger CM Kik SV Rangaka MX Zwerling A Oxlade O. Gamma Interferon Release Assays for Detection of Mycobacterium Tuberculosis Infection. Clin Microbiol Rev (2014) 27(1):3–20. 10.1128/CMR.00034-13 24396134 PMC3910908

[7] Meier T Eulenbruch HP Wrighton-Smith P Enders G Regnath T. Sensitivity of a New Commercial Enzyme-Linked Immunospot Assay (T SPOT-TB) for Diagnosis of Tuberculosis in Clinical Practice. Eur J Clin Microbiol Infect Dis (2005) 24(8):529–36. 10.1007/s10096-005-1377-8 16133410

[8] Chowdhury RR Vallania F Yang Q Angel CJL Darboe F Penn-Nicholson A. A Multi-Cohort Study of the Immune Factors Associated With M. Tuberculosis Infection Outcomes. Nature (2018) 560(7720):644–8. 10.1038/s41586-018-0439-x PMC6414221 30135583

[9] Berry MPR Graham CM McNab FW Xu Z Bloch SAA Oni T. An Interferon-Inducible Neutrophil-Driven Blood Transcriptional Signature in Human Tuberculosis. Nature (2010) 466(7309):973–7. 10.1038/nature09247 PMC3492754 20725040

[10] Joosten SA van Meijgaarden KE del Nonno F Baiocchini A Petrone L Vanini V. Patients With Tuberculosis Have a Dysfunctional Circulating B-Cell Compartment, Which Normalizes Following Successful Treatment. PloS Pathog (2016) 12(6):e1005687–1005624. 10.1371/journal.ppat.1005687 PMC4909319 27304615

[11] Janssens J-P Roux-Lombard P Perneger T Metzger M Vivien R Rochat T. Quantitative Scoring of an Interferon-Gamma Assay for Differentiating Active From Latent Tuberculosis. Eur Respir J (2007) 30(4):722–8. 10.1183/09031936.00028507 17537773

[12] Pollock KM Whitworth HS Montamat-Sicotte DJ Grass L Cooke GS Kapembwa MS. T-Cell Immunophenotyping Distinguishes Active From Latent Tuberculosis. J Infect Dis (2013) 208(6):952–68. 10.1093/infdis/jit265 PMC3749005 23966657

[13] Harari A Rozot V Enders FB Perreau M Stalder JM Nicod LP. Dominant TNF-α+ Mycobacterium Tuberculosis–Specific CD4+ T Cell Responses Discriminate Between Latent Infection and Active Disease. Nat Med (2011) 17(3):372–6. 10.1038/nm.2299 PMC6570988 21336285

[14] Goletti D Lee M-R Wang J-Y Walter N Ottenhoff THM. Update on Tuberculosis Biomarkers: From Correlates of Risk, to Correlates of Active Disease and of Cure From Disease. Respirology (2018) 23(5):455–66. 10.1111/resp.13272 29457312

[15] Adekambi T Ibegbu CC Cagle S Kalokhe AS Wang YF Hu Y. Biomarkers on Patient T Cells Diagnose Active Tuberculosis and Monitor Treatment Response. J Clin Invest (2015) 125(5):1827–38. 10.1172/JCI77990 PMC4598074 25822019

[16] Goletti D Lindestam Arlehamn CS Scriba TJ Anthony R Cirillo DM Alonzi T. Can We Predict Tuberculosis Cure? What Tools Are Available? Eur Respir J (2018) 52(5):1801089–1801060. 10.1183/13993003.01089-2018 30361242

[17] Cliff JM Kaufmann SHE McShane H van Helden P O'Garra A. The Human Immune Response to Tuberculosis and Its Treatment: A View From the Blood. Immunol Rev (2015) 264(1):88–102. 10.1111/imr.12269 25703554 PMC4368415

[18] Bournazos S Wang TT Dahan R Maamary J Ravetch JV. Signaling by Antibodies: Recent Progress. Annu Rev Immunol (2017) 35(1):285–311. 10.1146/annurev-immunol-051116-052433 28446061 PMC5613280

[19] Dryla A Prustomersky S Gelbmann D Hanner M Bettinger E Kocsis B. Comparison of Antibody Repertoires Against Staphylococcus Aureus in Healthy Individuals and in Acutely Infected Patient. Clin Vaccine Immunol (2005) 12(3):387–98. 10.1128/CDLI.12.3.387-398.2005 PMC1065207 15753252

[20] Stentzel S Sundaramoorthy N Michalik S Nordengrün M Schulz S Kolata J. Specific Serum IgG at Diagnosis of Staphylococcus Aureus Bloodstream Invasion Is Correlated With Disease Progression. J Proteomics (2015) 128(C):1–7. 10.1016/j.jprot.2015.06.018 26155744

[21] Varshney AK Wang X Aguilar JL Scharff MD Fries BC. Isotype Switching Increases Efficacy of Antibody Protection Against Staphylococcal Enterotoxin B-Induced Lethal Shock and Staphylococcus Aureus Sepsis in Mice. mBio (2014) 5(3):615–9. 10.1128/mBio.01007-14 PMC4056548 24917594

[22] Arnold JN Wormald MR Sim RB Rudd PM Dwek RA. The Impact of Glycosylation on the Biological Function and Structure of Human Immunoglobulins. Annu Rev Immunol (2007) 25(1):21–50. 10.1146/annurev.immunol.25.022106.141702 17029568

[23] de Jong SE Selman MHJ Adegnika AA Amoah AS van Riet E Kruize YCM. IgG1 Fc N-Glycan Galactosylation as a Biomarker for Immune Activation. Sci Rep (2016) 6(1):28207–28207. 10.1038/srep28207 PMC4910062 27306703

[24] Lin C-W Tsai M-H Li S-T Tsai T-I Chu K-C Liu Y-C. A Common Glycan Structure on Immunoglobulin G for Enhancement of Effector Functions. Proc Natl Acad Sci - PNAS (2015) 112(34):10611–6. 10.1073/pnas.1513456112 PMC4553773 26253764

[25] Ackerman ME Crispin M Yu X Baruah K Boesch AW Harvey DJ. Natural Variation in Fc Glycosylation of HIV-Specific Antibodies Impacts Antiviral Activity. J Clin Invest (2013) 123(5):2183–92. 10.1172/JCI65708 PMC3637034 23563315

[26] Ho C-H Chien R-N Cheng P-N Liu J-H Liu C-K Su C-S. Aberrant Serum Immunoglobulin G Glycosylation in Chronic Hepatitis B Is Associated With Histological Liver Damage and Reversible by Antiviral Therapy. J Infect Dis (2014) 211(1):115–24. 10.1093/infdis/jiu388 25015948

[27] Theodoratou E Thaçi K Agakov F Timofeeva MN Štambuk J Pučić-Baković M. Glycosylation of Plasma IgG in Colorectal Cancer Prognosis. Sci Rep (2016) 6(1):28098. 10.1038/srep28098 27302279 PMC4908421

[28] Ren S Zhang Z Xu C Guo L Lu R Sun Y. Distribution of IgG Galactosylation as a Promising Biomarker for Cancer Screening in Multiple Cancer Types. Cell Res (2016) 26(8):963–6. 10.1038/cr.2016.83 PMC4973333 27364686

[29] Di Sabatino A Biagi F Lenzi M Frulloni L Lenti MV Giuffrida P. Clinical Usefulness of Serum Antibodies as Biomarkers of Gastrointestinal and Liver Diseases. Digestive liver Dis (2017) 49(9):947–56. 10.1016/j.dld.2017.06.010 28733178

[30] Rombouts Y Ewing E van de Stadt LA Selman MHJ Trouw LA Deelder AM. Anti-Citrullinated Protein Antibodies Acquire a Pro-Inflammatory Fc Glycosylation Phenotype Prior to the Onset of Rheumatoid Arthritis. Ann Rheum Dis (2015) 74(1):234–41. 10.1136/annrheumdis-2013-203565 24106048

[31] Lee S-J Liang L Juarez S Nanton MR Gondwe EN Msefula CL. Identification of a Common Immune Signature in Murine and Human Systemic Salmonellosis. Proc Natl Acad Sci (2012) 109(13):4998–5003. 10.1073/pnas.1111413109 22331879 PMC3324033

[32] Demkow U Filewska M Michalowska-Mitczuk D Kus J Jagodzinski J Zielonka T. Heterogeneity of Antibody Response to Myobacterial Antigens in Different Clinical Manifestations of Pulmonary Tuberculosis. J Physiol Pharmacol an Off J Polish Physiol Soc (2007) 58 Suppl 5(Pt 1):117–27. 18204122

[33] Wang S Wu J Chen J Gao Y Zhang S Zhou Z. Evaluation of Mycobacterium Tuberculosis-Specific Antibody Responses for the Discrimination of Active and Latent Tuberculosis Infection. Int J Infect Dis (2018) 70:1–9. 10.1016/j.ijid.2018.01.007 29410147

[34] Lyashchenko K Colangeli R Houde M Al Jahdali H Menzies D Gennaro ML. Heterogeneous Antibody Responses in Tuberculosis. Infect Immun (1998) 66(8):3936–40. 10.1128/IAI.66.8.3936-3940.1998 PMC108457 9673283

[35] Alter G Ottenhoff THM Joosten SA. Antibody Glycosylation in Inflammation, Disease and Vaccination. Semin Immunol (2018) 39:102–10. 10.1016/j.smim.2018.05.003 PMC8731230 29903548

[36] Lu LL Chung AW Rosebrock TR Ghebremichael M Yu WH Grace PS. A Functional Role for Antibodies in Tuberculosis. Cell (2016) 167(2):1–26. 10.1016/j.cell.2016.08.072 27667685 PMC5526202

[37] Lu LL Das J Grace PS Fortune SM Restrepo BI Alter G. Antibody Fc Glycosylation Discriminates Between Latent and Active Tuberculosi. J Infect Dis (2020) 64:111–0. 10.1093/infdis/jiz643 PMC7661770 32060529

[38] Li H Wang X-x Wang B Fu L Liu G Lu Y. Latently and Uninfected Healthcare Workers Exposed to TB Make Protective Antibodies Against Mycobacterium Tuberculosis. Proc Natl Acad Sci USA (2017) 114(19):5023–8. 10.1073/pnas.1611776114 PMC5441709 28438994

[39] Chen T Blanc C Liu Y Ishida E Singer S Xu J. Capsular Glycan Recognition Provides Antibody-Mediated Immunity Against Tuberculosis. J Clin Invest (2020) 2015(6):1609. 10.1172/JCI128459 PMC7108924 31935198

[40] Zimmermann N Thormann V Hu B Köhler AB Imai Matsushima A Locht C. Human Isotype-Dependent Inhibitory Antibody Responses Against Mycobacterium Tuberculosis. EMBO Mol Med (2016) 8(11):1325–39. 10.15252/emmm.201606330 PMC5090662 27729388

[41] de Valliere S Abate G Blazevic A Heuertz RM Hoft DF. Enhancement of Innate and Cell-Mediated Immunity by Antimycobacterial Antibodies. Infect Immun (2005) 73(10):6711–20. 10.1128/IAI.73.10.6711-6720.2005 PMC1230956 16177348

[42] Roy E Stavropoulos E Brennan J Coade S Grigorieva E Walker B. Therapeutic Efficacy of High-Dose Intravenous Immunoglobulin in Mycobacterium Tuberculosis Infection in Mice. Infect Immun (2005) 73(9):6101–9. 10.1128/IAI.73.9.6101-6109.2005 PMC1231090 16113331

[43] Mattos AMM Chaves AS Franken KLMC Figueiredo BBM Ferreira AP Ottenhoff THM. Detection of IgG1 Antibodies Against Mycobacterium Tuberculosis DosR and Rpf Antigens in Tuberculosis Patients Before and After Chemotherapy. Tuberculosis (Edinburgh Scotland) (2016) 96(C):65–70. 10.1016/j.tube.2015.11.001 26786656

[44] Arias-Bouda LMP Kuijper S van der Werf A Nguyen LN Jansen HM Kolk AHJ. Changes in Avidity and Level of Immunoglobulin G Antibodies to Mycobacterium Tuberculosis in Sera of Patients Undergoing Treatment for Pulmonary Tuberculosis. Clin Diagn Lab Immunol (2003) 10(4):702–9. 10.1128/cdli.10.4.702-709.2003 PMC164257 12853408

[45] Chung AW Kumar MP Arnold KB Yu WH Schoen MK Dunphy LJ. Dissecting Polyclonal Vaccine-Induced Humoral Immunity Against HIV Using Systems Serology. Cell (Cambridge) (2015) 163(4):988–98. 10.1016/j.cell.2015.10.027 PMC5490491 26544943

[46] Gilpin C Korobitsyn A Migliori GB Raviglione MC Weyer K. The World Health Organization Standards for Tuberculosis Care and Management. Eur Respir J (2018) 51(3):1800098. 10.1183/13993003.00098-2018 29567724

[47] Tibshirani R. The Lasso Method for Variable Selection in the Cox Model. Stat Med (1997) 16(4):385–95. 10.1002/(SICI)1097-0258(19970228)16:4<385::AID-SIM380>3.0.CO;2-3 9044528

[48] Ackerman ME Das J Pittala S Broge T Linde C Suscovich TJ. Route of Immunization Defines Multiple Mechanisms of Vaccine-Mediated Protection Against SIV. Nat Med (2018) 24(10):1590–8. 10.1038/s41591-018-0161-0 PMC6482471 30177821

[49] Arnold KB Burgener A Birse K Romas L Dunphy LJ Shahabi K. Increased Levels of Inflammatory Cytokines in the Female Reproductive Tract Are Associated With Altered Expression of Proteases, Mucosal Barrier Proteins, and an Influx of HIV-Susceptible Target Cells. Mucosal Immunol (2016) 9(1):194–205. 10.1038/mi.2015.51 26104913

[50] Lau KS Juchheim AM Cavaliere KR Philips SR Lauffenburger DA Haigis KM. *In Vivo* Systems Analysis Identifies Spatial and Temporal Aspects of the Modulation of TNF-α-Induced Apoptosis and Proliferation by MAPK. Sci Signaling (2011) 4(165):ra16–6. 10.1126/scisignal.2001338 PMC3963028 21427409

[51] Chong I-G Jun C-H. Performance of Some Variable Selection Methods When Multicollinearity Is Present. Chemom Intell Lab Syst (2005) 78(1):103–12. 10.1016/j.chemolab.2004.12.011

[52] Alifano M Sofia M Mormile M Micco A Mormile AF Del Pezzo M. IgA Immune Response Against the Mycobacterial Antigen A60 in Patients With Active Pulmonary Tuberculosis. Respiration Int Rev Thorac Dis (1996) 63(5):292–7. 10.1159/000196563 8885002

[53] Abebe F Belay M Legesse M Franken KLMC Ottenhoff THM. IgA and IgG Against Mycobacterium Tuberculosis Rv2031 Discriminate Between Pulmonary Tuberculosis Patients, Mycobacterium Tuberculosis-Infected and non-Infected Individuals. PloS One (2018) 13(1):e0190989–0190919. 10.1371/journal.pone.0190989 PMC5786301 29373577

[54] Chandler KB Mehta N Leon DR Suscovich TJ Alter G Costello CE. Multi-Isotype Glycoproteomic Characterization of Serum Antibody Heavy Chains Reveals Isotype- and Subclass-Specific N-Glycosylation Profiles. Mol Cell Proteomics (2019) 18(4):686–703. 10.1074/mcp.RA118.001185 30659065 PMC6442369

[55] Konno N Sugimoto M Takagi T Furuya M Asano T Sato S. Changes in N-Glycans of IgG4 and Its Relationship With the Existence of Hypocomplementemia and Individual Organ Involvement in Patients With IgG4-Related Disease. PloS One (2018) 13(4):e0196163. 10.1371/journal.pone.0196163 29672582 PMC5908088

[56] Chung AW Ghebremichael M Robinson H Brown E Choi I Lane S. Polyfunctional Fc-Effector Profiles Mediated by IgG Subclass Selection Distinguish RV144 and VAX003 Vaccines. Sci Trans Med (2014) 6(228):228ra238–228ra238. 10.1126/scitranslmed.3007736 24648341

[57] Karagiannis P Gilbert AE Josephs DH Ali N Dodev T Saul L. IgG4 Subclass Antibodies Impair Antitumor Immunity in Melanoma. J Clin Invest (2013) 123(4):1457–74. 10.1172/JCI65579 PMC3613918 23454746

[58] Boes M. Role of Natural and Immune IgM Antibodies in Immune Responses. Mol Immunol (2000) 37(18):1141–9. 10.1016/S0161-5890(01)00025-6 11451419

[59] Lu LL Smith MT Yu KKQ Luedemann C Suscovich TJ Grace PS. IFN-γ-Independent Immune Markers of Mycobacterium Tuberculosis Exposure. Nat Med (2019) 25(6):997–87. 10.1038/s41591-019-0441-3 PMC6559862 31110348

[60] Chen T Blanc C Eder AZ Prados-Rosales R Souza ACO Kim RS. Association of Human Antibodies to Arabinomannan With Enhanced Mycobacterial Opsonophagocytosis and Intracellular Growth Reduction. J Infect Dis (2016) 214(2):300–10. 10.1093/infdis/jiw141 PMC4918826 27056953

[61] Kemna MJ Plomp R van Paassen P Koeleman CAM Jansen BC Damoiseaux JGMC. Galactosylation and Sialylation Levels of IgG Predict Relapse in Patients With PR3-ANCA Associated Vasculitis. EBioMedicine (2017) 17(C):108–18. 10.1016/j.ebiom.2017.01.033 PMC5360573 28169190

[62] Bondt A Selman MHJ Deelder AM Hazes JMW Willemsen SP Wuhrer M. Association Between Galactosylation of Immunoglobulin G and Improvement of Rheumatoid Arthritis During Pregnancy Is Independent of Sialylation. J Proteome Res (2013) 12(10):4522–31. 10.1021/pr400589m 24016253

[63] Wuhrer M Selman MHJ McDonnell LA Kumpfel T Derfuss T Khademi M. Pro-Inflammatory Pattern of IgG1 Fc Glycosylation in Multiple Sclerosis Cerebrospinal Fluid. J Neuroinflamm (2015) 12(236):235–5. 10.1186/s12974-015-0450-1 PMC4683913 26683050

[64] Yu X Prados-Rosales R Jenny-Avital ER Sosa K Casadevall A Achkar JM. Comparative Evaluation of Profiles of Antibodies to Mycobacterial Capsular Polysaccharides in Tuberculosis Patients and Controls Stratified by HIV Status. Clin Vaccine Immunol (2012) 19(2):198–208. 10.1128/CVI.05550-11 22169090 PMC3272928

[65] van Woudenbergh E Irvine EB Davies L de Kock M Hanekom WA Day CL. HIV Is Associated With Modified Humoral Immune Responses in the Setting of HIV/TB Coinfection. mSphere (2020) 5(3):603–19. 10.1128/mSphere.00104-20 PMC7380575 32434838

[66] Hussain R Poindexter RW Ottesen EA. Control of Allergic Reactivity in Human Filariasis. Predominant Localization of Blocking Antibody to the IgG4 Subclass. J Immunol (Baltimore Md 1950) (1992) 148(9):2731–7. 1573266

[67] Atmadja AK Atkinson R Sartono E Partono F Yazdanbakhsh M Maizels RM. Differential Decline in Filaria-Specific IgG1, IgG4, and IgE Antibodies in Brugia Malayi-Infected Patients After Diethylcarbamazine Chemotherapy. J Infect Dis (1995) 172(6):1567–72. 10.1093/infdis/172.6.1567 7594718

[68] Swierstra J Debets S de Vogel C Lemmens-den Toom N Verkaik N Ramdani-Bouguessa N. IgG4 Subclass-Specific Responses to Staphylococcus Aureus Antigens Shed New Light on Host-Pathogen Interaction. Infect Immun (2015) 83(2):492–501. 10.1128/IAI.02286-14 25404029 PMC4294233

[69] Siddiquee Z Smith RN Stone JR. An Elevated IgG4 Response in Chronic Infectious Aortitis Is Associated With Aortic Atherosclerosis. Modern Pathol (2015) 28: (11):1428–34. 10.1038/modpathol.2015.105 26336884

[70] Aalberse RC van der Gaag R van Leeuwen J. Serologic Aspects of IgG4 Antibodies. I. Prolonged Immunization Results in an IgG4-Restricted Response. J Immunol (Baltimore Md 1950) (1983) 130(2):722–6. 6600252

[71] Francis JN James LK Paraskevopoulos G Wong C Calderon MA Durham SR. Grass Pollen Immunotherapy: IL-10 Induction and Suppression of Late Responses Precedes IgG4 Inhibitory Antibody Activity. J Allergy Clin Immunol (2008) 121(5):1120–1125.e1122. 10.1016/j.jaci.2008.01.072 18374405

[72] Prodjinotho UF Hoerauf A Adjobimey T. IgG4 Antibodies From Patients With Asymptomatic Bancroftian Filariasis Inhibit the Binding of IgG1 and IgG2 to C1q in a Fc-Fc-Dependent Mechanism. Parasitol Res (2019) 118(10):2957–68. 10.1007/s00436-019-06451-2 PMC6754495 31485865

[73] Jackson KJL. Human Immunoglobulin Classes and Subclasses Show Variability in VDJ Gene Mutation Levelshuman Immunoglobulin Classes and Subclasses Show Variability in VDJ Gene Mutation Levels. Immunol Cell Biol (2014) 92(8):729–33. 10.1038/icb.2014.44Immunology 24913324

[74] Crescioli S Correa I Karagiannis P Davies AM Sutton BJ Nestle FO. IgG4 Characteristics and Functions in Cancer Immunity. Curr Allergy Asthma Rep (2016) 16(1):1–11. 10.1007/s11882-015-0580-7 26742760 PMC4705142

[75] Sousa AO Salem JI Lee FK Verçosa MC Cruaud P Bloom BR. An Epidemic of Tuberculosis With a High Rate of Tuberculin Anergy Among a Population Previously Unexposed to Tuberculosis, the Yanomami Indians of the Brazilian Amazo. Proc Natl Acad Sci USA (1997) 94(24):13227–32. 10.1073/pnas.94.24.13227 PMC24291 9371828

[76] Awoniyi DO Baumann R Chegou NN Kriel B Jacobs R Kidd M. Detection of a Combination of Serum IgG and IgA Antibodies Against Selected Mycobacterial Targets Provides Promising Diagnostic Signatures for Active TB. Oncotarget (2017) 8(23):37525–37. 10.18632/oncotarget.16401 PMC5514927 28415587

[77] Bothamley GH. Epitope-Specific Antibody Levels Demonstrate Recognition of New Epitopes and Changes in Titer But Not Affinity During Treatment of Tuberculosis. Clin Diagn Lab Immunol (2004) 11(5):942–51. 10.1128/CDLI.11.5.942-951.2004 PMC515269 15358657

