# Supplementary material for: Antibody Subclass and Glycosylation Shift Following Effective TB Treatment
Source: Front Immunol. 2021 Jul 5;12:679973. doi: 10.3389/fimmu.2021.679973 (PMC8287567; doi:10.3389/fimmu.2021.679973)
Supplement: Supplementary file 1 [file Image_1.pdf]

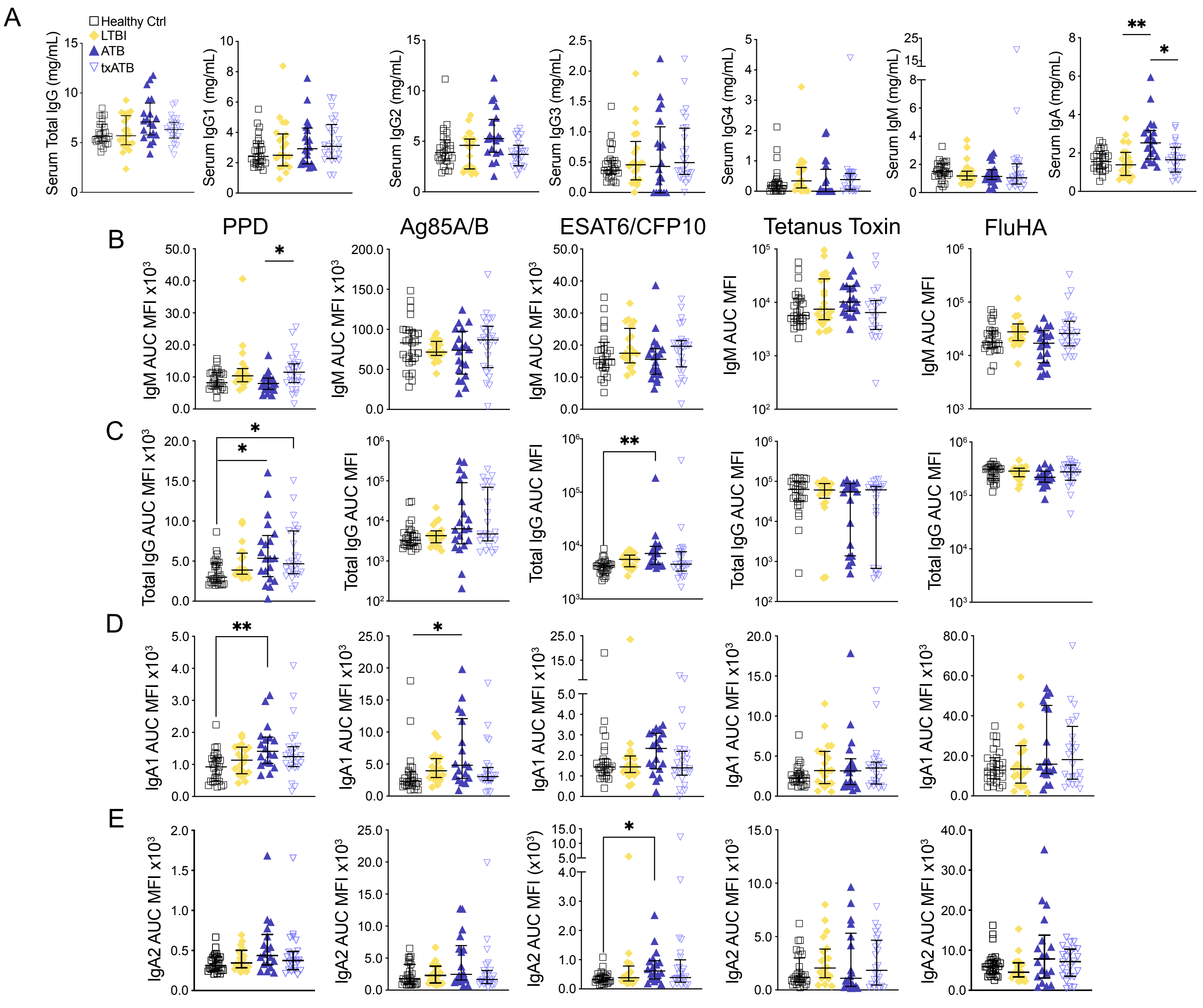

Supplemental Figure 1: ***Total plasma and antigen-specific isotype titers of cohort individuals*****. (A)** Total serum IgG1, IgG2, IgG3, IgG4, IgM, and IgA quantified with Luminex and total IgG quantified in an ELISA. Antigens PPD, Ag85A/B, ESAT6/CFP10, Tetanus Toxin and FluHA were used in a customized Luminex assay for to measure antigen-specific titers of **(B)** total IgM, **(C)** total IgG, **(D)** total IgA1, and **(E)** total IgA2. Plasma samples were diluted at 1:30, 1:100, 1:300, and 1:1000 and assayed for binding on antigen specific beads and area under the curve was generated for each antigen:isotype measure made per individual sample. Univariate plots **(A-E)** represent median and interquartile range of values measured in healthy control (n= 17, black open squares) LTBI (n=21, yellow diamonds), ATB (n=20, blue triangles), and txATB (n=23, periwinkle open triangles) individuals**.** Kruskall-Wallis test followed by Dunn's correction for multiple tests used to compare healthy control, LTBI, ATB, and txATB individuals: *p<0.05 and **p<0.01.

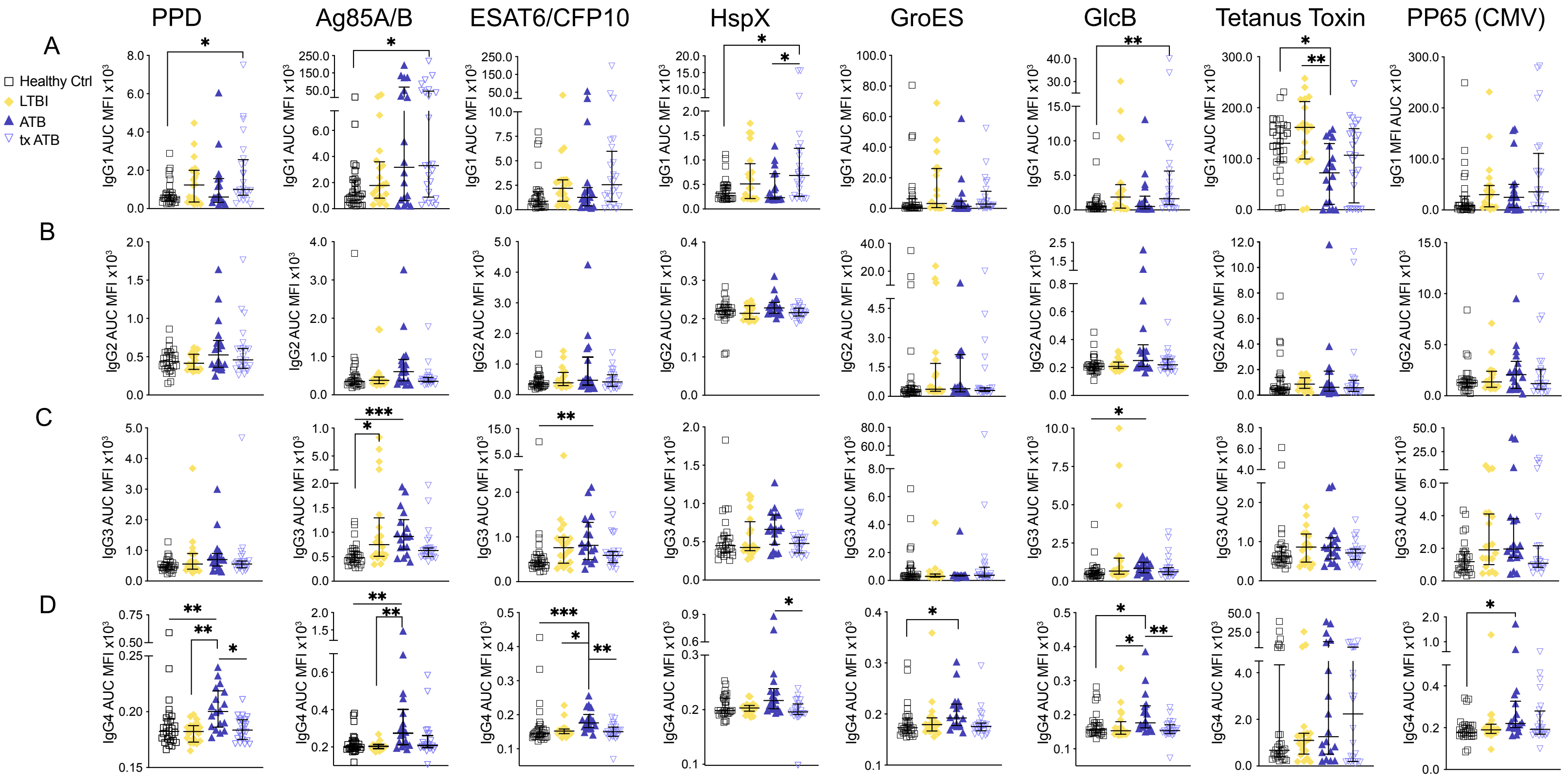

Supplemental Figure 2: ***Antigen-specific IgG subclass titers.*** Titer levels against antigens PPD, Ag85A/B, ESAT6/CFP10, HspX, GroES, GlcB, Tetanus Toxin and CMV-pp65 were measured via Luminex for **(A)** total IgG1, **(B)** total IgG2, **(C)** total IgG3, and **(D)** total IgG4. Plasma samples were diluted at 1:30, 1:100, and 1:300 and assayed for binding on antigen specific beads and area under the curve was generated for each antigen:isotype measure made per individual sample. Univariate plots **(A-D)** represent median and interquartile range of the measures from healthy control (n= 17, black open squares) LTBI (n=21, yellow diamonds), ATB (n=20, blue triangles), and txATB (n=23, periwinkle open triangles) individuals**.** Kruskall-Wallis test followed by Dunn's correction for multiple tests used to compare healthy control, LTBI, ATB, and txATB individuals: *p<0.05, **p<0.01, and ***p<0.005

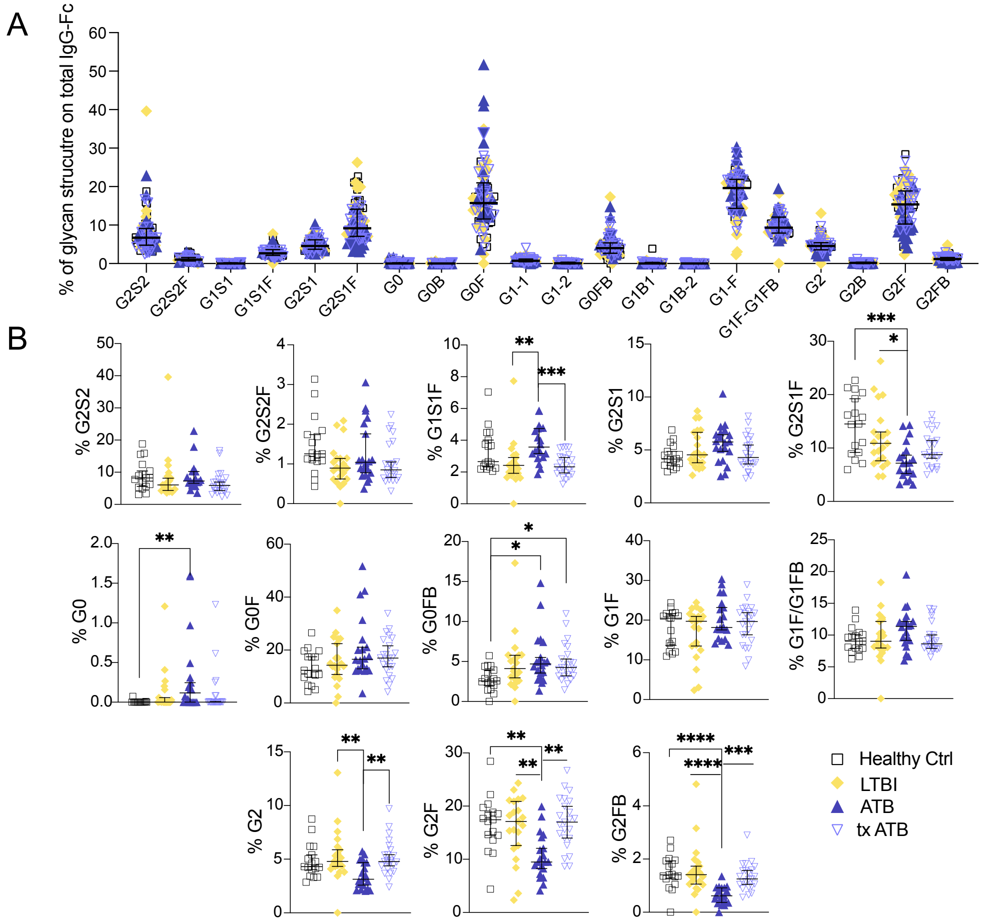

Supplemental Figure 3: ***Total IgG-Fc glycans.*** APTS-labelled N-linked glycan structures of the IgG-Fc measured by CE analysis. **(A)** Relative frequencies of measured glycans detectable by prelabelled standards demonstrate that a subset of detected glycans are differentially enriched across all individuals. Univariate plots **(B)** represent glycans measured with medians above 0% in healthy controls (n=17, black open squares), LTBI (n=21, yellow diamonds), ATB (n=20, blue triangles), or txATB (n=22, periwinkle open triangles) individuals**.** Data plotted as median and interquartile range of the measured values and statistical significance was calculated using Kruskall-Wallis test followed by Dunn's correction for multiple tests: *p<0.05, **p<0.01, ***p<0.005, and **** p<0.001.

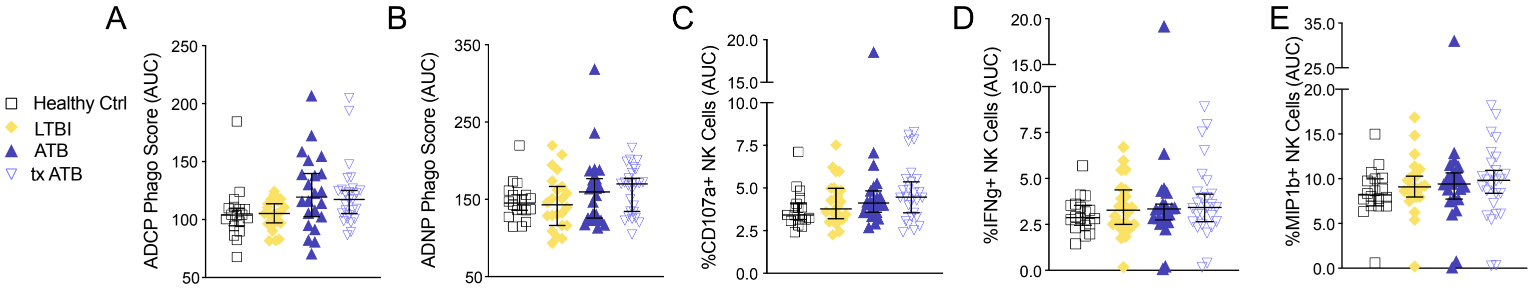

Supplemental Figure 4: ***Innate immune cell functional responses to plasma antibodies.*** Plasma from healthy control (n=17, black open squares), LTBI (n=21, yellow diamonds), ATB (n=20, blue triangles), and txATB (n=23, periwinkle open triangles) individuals were tested for FcR functional activity on innate immune cells. Biotin-PPD coated 1um streptavidin beads were combined with plasma at 1:30, 1:100, and 1:300 dilutions to form immune complexes; these complexes were combined with THP1 cells to measure antibody-dependent cellular phagocytosis (ADCP) **(A)**. Neutrophils isolated from healthy donors were also combined with immune complexes to measure antibody-dependent neutrophil phagocytosis (ADNP) **(B)**, n=6 replicates averaged for a composite AUC phago score. The ability of these plasma samples to induce NK cell degranulation and activation of PPD-specific antibodies bound to ELISA plates was determined by the frequencies of **(C)** CD107a+ **(D)** IFNγ+ and **(E)** MIP1β+ NK cells. Data in (**C-D)** represent an average of signal measured in a minimum of 3 independent donors. AUC for each donor test was generated from plasma dilutions of 1:30, 1:100, and 1:300. No statistically significant difference was found between healthy controls, LTBI, ATB, and txATB using Kruskall-Wallis test followed by Dunn's correction for multiple tests.

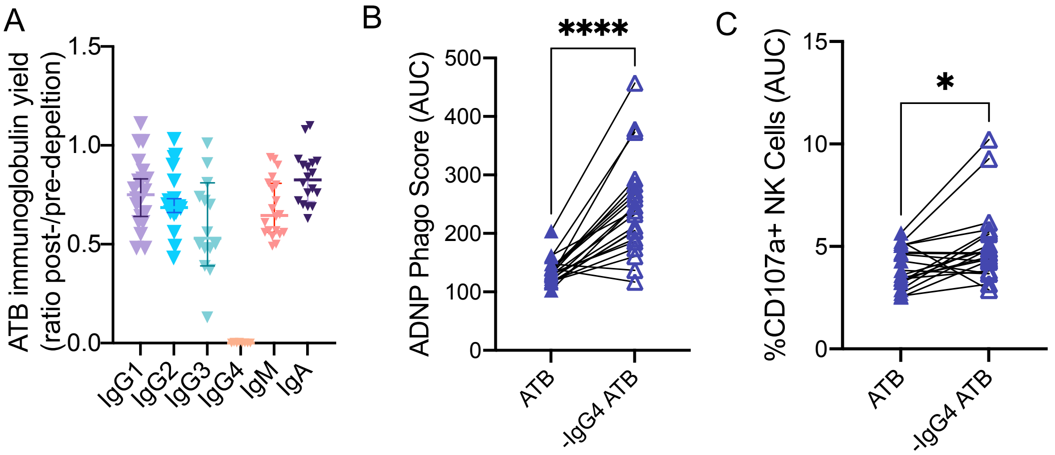

Supplemental Figure 5: ***IgG4 depletion in ATB serum increases antibody function on human neutrophils and NK cells.*** Serum was depleted of IgG4 and tested for antibody mediated function in Neutrophils and NK Cells. (**A**) Recovery of antibody isotypes and subclasses following IgG4 depletion. **(B)** PPD-specific antibodies in serum with and without IgG4 depletion were tested for function in an Antibody-Dependent Neutrophil Phagocytosis (ADNP), data are representative of n=2 independent healthy neutrophil donors. **(C)** PPD-specific antibodies in serum with and without IgG4 was tested for the CD107a expression on NK cells (degranulation induction). Data are representative of n=3 independent healthy NK cell donors. Scores for IgG4 depleted samples are corrected for loss of totaled immunoglobulins following depletion. ***S***tatistical significance was calculated using a Mann-Whitney : *$p<0.05$ and **** $p<0.001$.

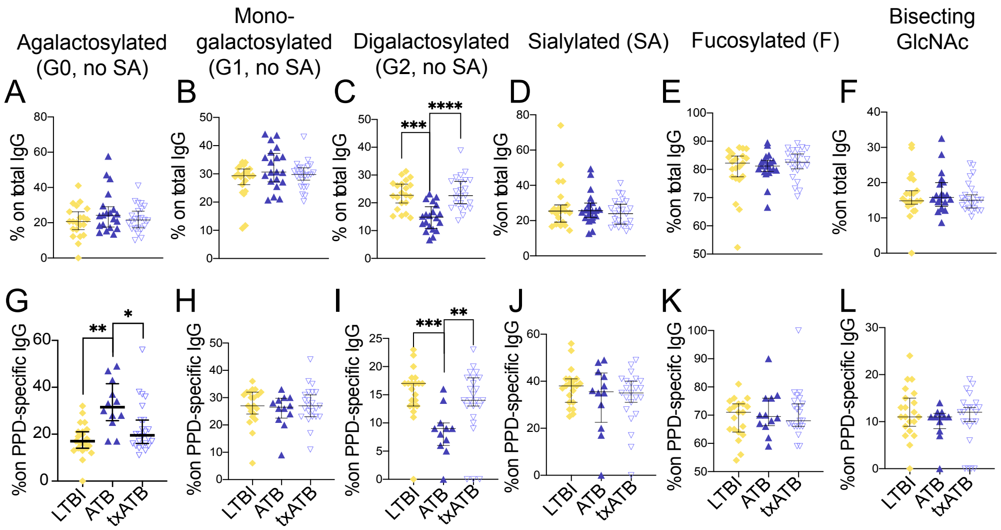

Supplemental Figure 6: ***Antigen-specific IgG glycans are enriched for additional features distinct from total IgG-Fc glycans that distinguish LTBI and txATB individuals from ATB.*** Individual glycan structures identified by CE on bulk IgG-Fc **(A-F)** and PPD-specific IgG-Fc **(G-L)** were totaled into the major categories of **(A ,G)** agalactosylated structures (G0; all structures lacking galactose), **(B, H)** mono-galactosylated structures (G1; all structures containing a single galactose), **(C, I)** digalactosylated structures (G2; all structures containing two galactose structures), **(D, J)** sialylated structures (SA; all structures with at least one sialic acid), **(E, K)** fucose (all structures containing fucose), and **(F, L)** bisecting N-acetylglycosamine (GlcNAC-B; all structures containing a bisecting GlcNAc). Univariate plots **(A-F)** represent LTBI (n=21, yellow diamonds), ATB (n=20, blue triangles), and txATB (n=23, periwinkle open triangles) individuals. Univariate plots of antigen- specific glycans **(G-L)** represent a subset of the cohort; LTBI (n=18), ATB (n=11), and txATB (n=26). Statistical significance between LTBI, ATB and txATB were calculated using Kruskall-Wallis test followed by Dunn's correction for multiple tests: *p<0.05, **p<0.01, ***p<0.0005, and **** p<0.0001.
